# Frequent Use of Premenopausal Progestin in Women With Prior Preeclampsia

**DOI:** 10.1210/clinem/dgae677

**Published:** 2024-10-08

**Authors:** Johanna M Joensuu, Olavi Ylikorkala, Minttu Venetkoski, Mika Gissler, Hanna Savolainen-Peltonen, Tomi S Mikkola

**Affiliations:** Department of Obstetrics and Gynecology, University of Helsinki and Helsinki University Hospital, Helsinki FI-00290, Finland; Department of Obstetrics and Gynecology, University of Helsinki and Helsinki University Hospital, Helsinki FI-00290, Finland; Department of Obstetrics and Gynecology, University of Helsinki and Helsinki University Hospital, Helsinki FI-00290, Finland; Department of Knowledge Brokers, Finnish Institute for Health and Welfare, Helsinki FI-00271, Finland; Academic Primary Health Care Centre, Region Stockholm, Stockholm 45436, Sweden; Department of Molecular Medicine and Surgery, Karolinska Institutet, Stockholm 17177, Sweden; Department of Obstetrics and Gynecology, University of Helsinki and Helsinki University Hospital, Helsinki FI-00290, Finland; Department of Obstetrics and Gynecology, University of Helsinki and Helsinki University Hospital, Helsinki FI-00290, Finland

**Keywords:** premenopause, menopausal hormone therapy, progestogen, abnormal menstruation, intrauterine device, menstrual cycle

## Abstract

**Context:**

Women with a history of preeclamptic pregnancy are predisposed to later occlusive vascular diseases.

**Objective:**

We compared the use of cyclic progestins or levonorgestrel-releasing intrauterine device (LNG-IUD) for treatment of menstrual cycle abnormalities between premenopausal women with and without a prior preeclamptic pregnancy.

**Methods:**

Register-based cohort study during 1994 to 2019 of oral progestin or LNG-IUD in Finnish women with (n = 31 688) and without (n = 91 726) prior preeclampsia in 1969 to 1993. Cyclic progestin or LNG-IUD use and its association with future use of menopausal hormone therapy (MHT).

**Results:**

Women with prior preeclampsia had used cyclic progestins more often (23.5% vs 9.1%; *P* < .001) and initiated the use at younger ages (41.8 years, SD 6.3 vs 45.9 years, 3.1; *P* < .001) than control women. Also, LNG-IUD was inserted more frequently (*P* < .001) in women with prior preeclampsia (9.3%) than in controls (4.7%). Cyclic progestin or LNG-IUD use was accompanied by significant 37% to 90% elevations in future MHT use.

**Conclusion:**

Increased use of cyclic progestins and LNG-IUD in premenopausal women with a history of preeclamptic pregnancy can be seen as indirect evidence of earlier onset of ovulatory dysfunction. This may contribute to the elevated risk of endometrial cancer in these women. Our findings may indicate an additional late sequela of preeclamptic pregnancy.

Preeclampsia is a systemic vascular disorder that complicates 2% to 5% of pregnancies worldwide. It has been well-established that preeclampsia is accompanied by a 2- to 3-fold elevation in lifelong risk for various cardiovascular diseases (CVDs) (1-3) and chronic renal disorders (4, 5). A 2-fold rise in the risk of diabetes is also associated with a history of preeclampsia (6), along with reduced thyroid function (7) and increased sympathetic activity in later life (8). Severe menopausal hot flashes may also occur more commonly in women with prior preeclampsia (9, 10), suggesting a link between preeclamptic history and menopausal estrogen deficiency.

Important in the genesis of preeclampsia and subsequent atherosclerosis are vascularization defects either in the placenta or in arterial walls (11-13). Ovulation and subsequent formation of corpus luteum require delicate hormonal interactions and readily adaptive ovarian blood flow and active angiogenesis in ovarian cells (14, 15). Thus, we speculated that if prior preeclampsia had an impact on ovarian function, it would most likely manifest as anovulatory cycles or inadequate corpus luteum formations. These phenomena are characterized in menopausal transition, manifesting clinically as irregular or abundant menstruations (16). In clinical practice, these symptoms are typically treated with cyclic progestin therapy (CPT) courses or by inserting a levonorgestrel-releasing intrauterine device (LNG-IUD). To assess the possible impact of prior preeclampsia on premenopausal ovarian function, we compared use of CPT or LNG-IUD between premenopausal women with and without a history of preeclamptic pregnancy.

## Materials and Methods

We followed women who were included in our national controlled cohort study of the impact of prior preeclampsia on CVD risks (17). Briefly, 31 688 women with established preeclampsia and 91 726 control women with a history of normotensive pregnancy were traced from the Hospital Discharge Register in 1969 to 1993. The diagnoses, according to international criteria (ICD-8 and ICD-9), were classified as preeclampsia without severe features (n = 25 814, now “mild”), preeclampsia with severe features (n = 4867, now “severe”), and eclampsia (n = 1007) (17). At inclusion, both the average ages at inclusion (mean 28.6 years [SD = 5.7], vs 28.6 years [SD = 5.7]) and the proportions of women older than 40 years were comparable for women with preeclampsia (3.5%) and control women (3.4%, *P* = .63) (17). The parity in the preeclamptic cohort (mean 2.6 [SD = 1.5]) was higher (*P* < .001) than that in control cohort (2.5 [SD = 1.3], *P* < .001). Prior preeclampsia was accompanied by a mean 1.5-fold rise in future CVD risk already at the age of 62 years, which was the mean age at the end of the CVD follow-up.

In this study, we followed these 2 study populations for the use of CPT or LNG-IUD with the aid of the National Reimbursement Register in 1994 to 2019. CPT and LNG-IUD are available with a prescription, but only the CPT or LNG-IUD purchases that are for medical indications marked on the prescription are reimbursed (cycle irregularities, heavy menstrual bleedings), and, thus, these purchases are entered into the Register. In contrast, CPT or LNG-IUD used for postponing menstruation or sole contraception are not reimbursed and, thus, they are not entered into the Register. For menstrual cycle abnormalities, the Finnish treatment guidelines advise the use of various progestins for 10 to 14 days starting on cycle day 15, usually for 3 consecutive menstrual cycles. Various progestins are administered orally and micronized progesterone either orally or vaginally. All oral or vaginal progestins were analyzed as a single group. Daily doses of these progestins are 5 to 10 mg and that of progesterone 100 to 200 mg orally or vaginally. The women in both cohorts were followed for CPT use from 1994 to 2019. The follow-up was discontinued at 52 years of age because Finnish women go into menopause at the mean age of 51 years (18). We also traced from the registry women with LNG-IUD purchases for medical indications such as control of irregular or abundant menstruation in women older than 40 years. LNG-IUD purchase resulted in discontinuation of the follow-up since most women with LNG-IUD are in amenorrhea. The follow-up was also terminated if the woman purchased any menopausal hormone therapy (MHT) regimen, emigrated, or died, or at the end of 2019 (Table 1). In total, 1.54 million woman-years accumulated with a mean follow-up of 13.4 (7.1) years for women with prior preeclampsia and 13.7 (7.5) years for control women.

**Table 1.** Baseline characteristics of study populations collected in 1969 to 1993 and followed for the use of CPT and LNG-IUD in 1994 to 2019

	Women with history of preeclampsia (n = 31 688)	Women without history of preeclampsia (n = 91 726)	Sig.
Age at diagnosis of preeclampsia, years (SD)	28.6 (5.7)	28.6 (5.7)	*P* = .86
**Type of preeclampsia**			
Mild	25 814 (81.5%)	N/A	
Severe	4867 (15.4%)	N/A	
Eclampsia	1007 (3.2%)	N/A	
**Region**			
Southern or western	22 340 (70.5%)	64 874 (70.7%)	*P* = .45
Eastern or northern	9348 (29.5%)	26 852 (29.3%)	
Age at beginning of follow-up in 1994, years	38.1 (8.2)	38.0 (8.1)	*P* = .31
Age at end of follow-up in 2019, years	51.6 (1.6)	51.7 (1.2)	*P* = .31
**Termination of follow-up due to**			
Insertion of LNG-IUD	2936 (9.3%)	4280 (4.7%)	*P* < .001
Start of MHT <52 years	5880 (18.6%)	17 144 (18.7%)	
Emigration	131 (0.4%)	1140 (1.2%)	
Death	675 (2.1%)	1798 (2.0%)	
Reaching 52 years	20 803 (65.6%)	63 004 (68.7%)	
Age <52 years at end of 2019	1263 (4.0%)	4360 (4.8%)	
Duration of follow-up, years	13.4 (7.1)	13.7 (7.5)	*P* < .001
Woman-years followed	398 205	1 174 978	

Abbreviations: CPT, cyclic progestin therapy; LNG-IUD, levonorgestrel-releasing intrauterine device; MHT denotes menopausal hormone therapy

We also assessed whether CPT or LNG-IUD was associated with the severity of prior preeclampsia, as indicated by its occurrence before the age of 24 years or repeatedly or by its progressing to eclampsia. We further speculated that deficiency of ovarian blood flow, reflected in CPT or LNG-IUD use, might occur in the same women who later had occlusive events in large arteries. Therefore, we related the use of CPT or LNG-IUD to the occurrence of ischemic heart disease (n = 6 857), myocardial infarction (n = 2 152), or stroke (n = 5 211) (17).

### Statistical Analysis

We compared the proportions of CPT, LNG-IUD, and MHT users with and without a history of preeclampsia with the chi-square test. The impact of preceding preeclamptic characteristics with CPT or LNG-IUD use was evaluated with odds ratios and 95% CIs. The significance of the difference in the age at initiation of CPT or MHT or insertion of LNG-IUD was analyzed with Student's or Welch's t-test depending on the equality of variances.

### Permissions

The study protocol was approved by the research committee of the Helsinki University Hospital (HUS/222/2021) and by the Finnish Social and Health Data Permit Authority Findata (THL/3683/14.02.00/2020). Study subjects were traced with individual social security codes, but they were anonymized for further analyses, and subjects' identities were never revealed. Since this was a register-based study, patient consent was not needed.

## Results

**Figure 1.**
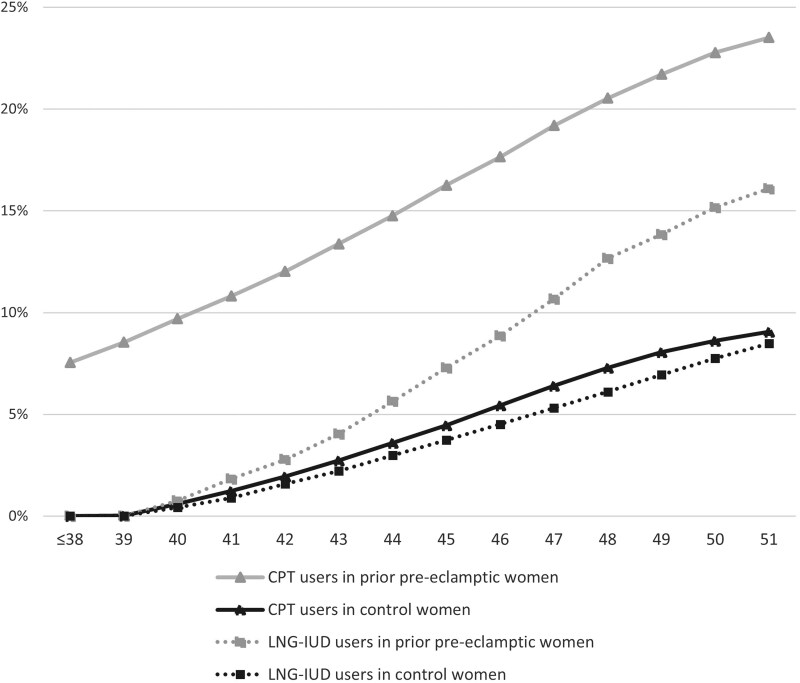
Cumulative proportions (%) of women with and without a history of pre-eclampsia who had used cyclic progestin therapy (CPT) at age 38 to 51 years and women with levonorgestrel-releasing intrauterine device (LNG-IUD) insertion at age 40 to 51 years. The groups differ significantly from each other.

Women with or without prior preeclamptic pregnancy were on average 38 years when they were included in the CPT or LNG-IUD follow-up (Table 1 and Fig. 1). The women were followed for a mean of 13.6 years, and they were a mean age of 51.7 years at termination of the study. If not before, the follow-up terminated at the age of 52 years, and approximately 95% of women had reached this age before study cessation.

The LNG-IUD was inserted more often (9.3% vs 4.7%; *P* < .001) in women with prior preeclampsia than in controls (Fig. 1), and this terminated the follow-up. Other reasons for follow-up termination were similar in both groups (Table 1). Both groups were followed for approximately 13.5 years, yielding 0.4 million follow-up years for women with prior preeclampsia and 1.2 million years for controls (Table 1).

Altogether, 6 different progestins had been used, most commonly norethisterone acetate (41.4%), followed by dydrogesterone (28.6%) and medroxyprogesterone acetate (15.3%). Other less frequently used progestins comprised megestrol acetate, lynestrenol, and micronized progesterone.

Compared with control women, women with prior preeclampsia had used CPT courses 2.3 times more frequently (Table 2). These CPT courses took place at a younger age and more often repeatedly, particularly before LNG-IUD insertion (Table 2). The need for first CPT or insertion of LNG-IUD appeared 2.3 years earlier (*P* < .001) in women with prior preeclampsia (Table 2). Furthermore, insertion of LNG-IUD was preceded by more frequent and more often repeated CPT use (Table 2).

**Table 2.** Patterns of CPT and LNG-IUD in women with and without a history of preeclamptic pregnancy

		Women with history of preeclampsia (n = 31 688)	Women without history of preeclampsia (n = 91 726)	
CPT use	Yes	7451 (23.5%)	8316 (9.1%)	OR 3.08 (95% CI 2.98, 3.19)
Age at first CPT use, years	Mean (SD)	41.8 (6.3)	45.9 (3.1)	*P* < .001
Years between the index day and first CPT use/insertion of LNG-IUD	Mean (SD)	13.9 (6.4)	16.2 (5.8)	*P* < .001
Number of CPT courses	Mean (SD)	3.0 (4.4)	2.7 (3.2)	*P* < .001
LNG-IUD	Yes	2936 (9.3%)	4280 (4.7%)	*P* < .001
Age at insertion of LNG-IUD	Mean (SD)	47.6 (4.3)	48.4 (4.8)	*P* < .001
CPT before LNG-IUD	1	333 (11.3%)	308 (7.2%)	*P* < .001
	2-3	196 (6.7%)	171 (4.0%)	
	>3	125 (4.3%)	98 (2.3%)	

Abbreviations: CPT, cyclic progestin therapy; LNG-IUD, levonorgestrel-releasing intrauterine device; OR, odds ratio.

The use of CPT was followed more often by MHT (OR 1.90; 95% CI 1.80-2.00), which was started at a younger age (49.5 [3.6] vs 50.8 [3.4] years, *P* < .001). Also, LNG-IUD was associated with more common MHT use (OR 1.39; 1.29-1.50), which was initiated at an older age (50.9 [5.1] vs 50.3 [3.6] years, *P* < .001) (Table 3).

**Table 3.** Relationship between CPT or LNG-IUD use and later MHT

	MHT	no MHT	OR (95% CI)
Women without CPT or LNG-IUD use n = 21 956	5875 (26.8%)	16 081 (73.2%)	1.00 (ref.)
Women with CPT n = 7451	3139 (42.1%)	4312 (57.9%)	1.99 (1.89-2.10)
Women with LNG-IUD n = 2935	1109 (37.8%)	1826 (62.2%)	1.66 (1.53-1.80)

Abbreviations: CPT, cyclic progestin therapy; levonorgestrel-releasing intrauterine device, LNG-IUD; MHT, menopausal hormone therapy; OR, odds ratio

The CPT and LNG-IUD uses were comparable in women with mild (n = 8033) or severe preeclampsia (n = 1386), or with eclampsia (n = 313) (OR 0.98; 0.86-1.12). If preeclampsia had occurred before the age of 24 years, indicating a potential for its severe form, the use of either CPT or LNG-IUD was increased (OR 1.20; 1.14-1.27). Moreover, CPT or LNG-IUD use failed to predict CVD events in large arteries.

## Discussion

We showed that premenopausal women with prior preeclampsia use CPT regimens more often, at a younger age, and more frequently than control women. Also, these women were treated with LNG-IUD more often than controls. These findings indicate irregular or abundant menstruation in women with prior preeclampsia since these are the primary indications for CPT or LNG-IUD use in this age group.

Cycle aberrations can occur at any age, but they become more common during menopausal transition as a result of anovulation and luteal deficiencies (16). Indeed, 44% of premenopausal women (mean age 47 years) with short cycle intervals (<21 days) and 65% of women with long cycle intervals (>36 days) proved to be anovulatory based on serial hormone assays (16). Even had ovulations occurred, progesterone supply was likely inadequate to support the endometrium. Besides cycle changes, this also leads to abundant menstruation or even menorrhagia. Thus, our data on frequent use of CPT and LNG-IUD may imply an adverse effect of prior preeclampsia on ovulatory function during menopausal transition.

The mechanisms by which prior preeclampsia may lead to this phenomenon are unknown, but we offer 2 explanations. First, prior preeclampsia predisposes to 2- to 3-fold lifelong risks of occlusive diseases in large arteries (1-3, 19), and this was seen also in our original cohort study (17). It is possible that ovarian arteries resemble larger arteries in this regard and are prone to vasoconstrictive and even occlusive changes. This may manifest first at ovulation, when ovarian blood flow should rapidly increase (14). This theory is supported by data that diminished ovarian blood flow after previous embolization of uterine artery was accompanied by abnormal menstrual cycles at approximately 45 years of age (20). On the other hand, the theory may be argued by our data since CPT or LNG-IUD use failed to predict future events in large arteries. A second and more likely explanation involves angiogenesis. Preeclampsia is characterized by poor development of spiral arteries in early placenta and insufficient vascularization (12, 13). If a similar failure in vascularization, probably derived inherently, occurred in highly vascularized growing follicles or corpus luteum, anovulation and luteal deficiencies would ensue (15, 21). It is noteworthy that preeclampsia-related late adverse vascular changes in large arteries appear approximately 1 decade earlier than in women with uneventful obstetric history (2, 3), and this timing fits well with menopausal transition. Poor angiogenesis may be the result of changes in reproductive hormone levels, although they were normal within the first 5 years after preeclamptic pregnancy (22), typically at younger ages than studied here. In this scenario, luteinizing hormone could be a key factor because it promotes angiogenesis potently in ovarian endothelial cells (21). Future laboratory studies are needed to ascertain the role of reproductive hormones in possible ovulatory dysfunction in these women.

Based on the whole cohort, we have reported that women with prior preeclampsia initiated MHT at similar ages (49.9 years) and used it equally as often (ca. 31%) as women without prior preeclampsia (17). The higher MHT use rate and initiation at a younger age in women with CPT may derive partly from the fact that they had consistent contact with their physicians, most often gynecologists, and that they had become accustomed to hormone use. It is also possible that some MHT prescriptions in previous CPT users had been given to control menstrual cycle aberrations rather than vasomotor symptoms. This may also be supported by our data that women with LNG-IUD started MHT use later because these women are typically in amenorrhea. We interpret these data as indirect evidence that prior preeclampsia does not predispose to hypoestrogenic states or premature menopause.

Some limitations of our study must be addressed. First, and most importantly, we admit that CPT use and LNG-IUD insertion are only indirect markers for ovulatory dysfunction and that our findings must be confirmed with careful studies on the circulating levels of reproductive hormone, ovarian morphology, and endometrial histology. Second, we could not control for the role of polycystic ovary syndrome (PCOS), a condition characterized by menstrual cycle irregularities and, if pregnancy ensues, increased risk of preeclampsia and related adverse pregnancy outcomes (11, 23). The prevalence of PCOS varies between 6% and 10% depending on the diagnostic criteria (24), and assisted reproduction methods, such as in vitro fertilization, are often applied as infertility treatment for these women (25). The small risk elevation (29%) for preeclampsia in subjects with PCOS in a recent large study (26) and the relative infertility of these women may imply that the proportion of women with PCOS in our cohorts with proven fertility is unlikely to be high enough to result in a major bias. Third, one can argue that CPT could have been prescribed for postponing menstruations. These CPT purchases are made for nonmedical indications, and, therefore, as well as hormonal contraceptives they are not entered into the Reimbursement Register. Regarding genuine menorrhagia, it is treated in Finland most commonly with an insertion of LNG-IUD, and these women were included in the follow-up. The remainder of women with heavy menstruations may have been treated with fibrinolysis inhibitors, endometrial ablation, or hysterectomy. The incidence of hysterectomy has decreased markedly, and the mean age of women undergoing the procedure has risen from 51 to 56 years during our follow-up (27). Thus, the majority of hysterectomized women did not produce follow-up years in our study because the follow-up was terminated at 52 years of age (27). Thus, women with hormonal contraception or hysterectomy are primary sources of errors, but their impact is small and could only dilute our conclusions.

The study has several strengths. First, the study populations were large. Second, the study subjects were collected evenly throughout Finland such that they represent well the national CPT and LNG-IUD usage policy. Third, the National Reimbursement Register is reliable and has been used in many previous studies (28-30). Fourth, 95% of the study subjects underwent menopausal transition during the follow-up, and, thus, could present symptoms linked to ovulatory dysfunction.

The clinical significance of our findings can only be speculated. We studied premenopausal women, and, thus, our data cannot be applied to assess fertility, but it is known that a history of preeclampsia does not decrease fertility within the first years after preeclamptic pregnancy (31). Repeated anovulations result in unopposed hyperestrogenic endometrium, which precedes endometrial hyperplasia and cancer. Indeed, based on the meta-analysis of 7 studies, preeclampsia was accompanied by a 34% rise in the risk of endometrial premalignant and malignant changes (32). In contrast, the risk of breast cancer, the most common hormone-sensitive cancer, is unaffected by a history of preeclamptic pregnancy (33).

To sum up, prior preeclamptic pregnancy may predispose to an early onset of ovulatory dysfunction during menopausal transition. Thus, ovulatory dysfunction could be an additional late adverse effect of preeclampsia.

## Data Availability

Restrictions apply to the availability of all data analyzed in this study because they were used under regional license.

## Abbreviations

CVD: cardiovascular disease
CPT: cyclic progestin therapy
LNG-IUD: levonorgestrel-releasing intrauterine device
MHT: menopausal hormone therapy’
PCOS: polycystic ovary syndrome
